# Use of a Recombinant Cysteine Proteinase from *Leishmania (Leishmania) infantum chagasi* for the Immunotherapy of Canine Visceral Leishmaniasis

**DOI:** 10.1371/journal.pntd.0002729

**Published:** 2014-03-13

**Authors:** Josie Haydée Lima Ferreira, Lucilene dos Santos Silva, Ieda Maria Longo-Maugéri, Simone Katz, Clara Lúcia Barbiéri

**Affiliations:** 1 Departamento de Microbiologia, Imunologia e Parasitologia, Escola Paulista de Medicina, Universidade Federal de São Paulo, São Paulo, São Paulo, Brazil; 2 Departamento de Parasitologia e Microbiologia, Centro de Ciências da Saúde, Universidade Federal do Piauí, Teresina, Piauí, Brazil; 3 Centro de Ciências Agrárias, Universidade Federal do Piauí, Teresina, Piauí, Brazil; Yale School of Public Health, United States of America

## Abstract

**Background:**

A recombinant cysteine proteinase from *Leishmania (Leishmania) infantum chagasi* (rLdccys1) was previously shown to induce protective immune responses against murine and canine visceral leishmaniasis. These findings encouraged us to use rLdccys1 in the immunotherapy of naturally infected dogs from Teresina, Piauí, a region of high incidence of visceral leishmaniasis in Brazil.

**Methodology/Principal Findings:**

Thirty naturally infected mongrel dogs displaying clinical signs of visceral leishmaniasis were randomly divided in three groups: one group received three doses of rLdccys1 in combination with the adjuvant *Propionibacterium acnes* at one month interval between each dose; a second group received three doses of *P. acnes* alone; a third group received saline. The main findings were: 1) dogs that received rLdccys1 with *P. acnes* did not display increase of the following clinical signs: weight loss, alopecia, onychogryphosis, cachexia, anorexia, apathy, skin lesions, hyperkeratosis, ocular secretion, and enlarged lymph nodes; they also exhibited a significant reduction in the spleen parasite load in comparison to the control dogs; 2) rLdccys1-treated dogs exhibited a significant delayed type cutaneous hypersensitivity elicited by the recombinant antigen, as well as high IgG2 serum titers and low IgG1 serum titers; sera from rLdccys1-treated dogs also contained high IFN-γ and low IL-10 concentrations; 3) control dogs exhibited all of the clinical signs of visceral leishmaniasis and had low serum IgG2 and IFN-γ levels and high concentrations of IgG1 and IL-10; 4) all of the dogs treated with rLdccys1 were alive 12 months after treatment, whereas dogs which received either saline or *P. acnes* alone died within 3 to 7 months.

**Conclusions/Significance:**

These findings illustrate the potential use of rLdccys1 as an additional tool for the immunotherapy of canine visceral leishmaniasis and support further studies designed to improve the efficacy of this recombinant antigen for the treatment of this neglected disease.

## Author Summary

Visceral leishmaniasis (VL) is an important public health problem and dogs are the main domestic reservoirs of zoonotic VL which has resulted in an annual incidence of 40,100–75,500 new human cases. Because canine VL chemotherapy is limited by the low efficacy of drugs currently used for human VL treatment, immunotherapy may provide a viable alternative. We used a recombinant cysteine proteinase from *L. (L.) infantum chagasi*, rLdccys1, in combination with the adjuvant *P. acnes* for the treatment of naturally infected mongrel dogs from Teresina, Pauí a state in Brazil that has a high incidence of VL. Dogs treated with rLdccys1 showed a significant delayed type hypersensitivity reaction against the recombinant antigen and displayed high serum concentrations of IgG2 and IFN-γ and low concentrations of IgG1 and IL-10. Immunotherapy with rLdccys1 resulted in no increase of the clinical signs of canine VL and an extensive reduction of spleen parasite load. Furthermore, all of the dogs treated with rLdccys1 survived for at least 12 months after treatment, whereas those that received either saline or *P. acnes* alone died within 3 to 7 months. These findings support the potential of rLdccys1 immunotherapy as an additional option for the treatment of canine VL.

## Introduction

Zoonotic visceral leishmaniasis (VL) is caused by *Leishmania (Leishmania) infantum chagasi* in Mediterranean, Middle-East, Asian countries, and Latin America and dogs are the main domestic reservoirs of this zoonosis which has resulted in an annual incidence of 40,100–75,000 new human cases [1], [2]. A high human VL incidence has been reported in Brazil mainly due to disease urbanization as a consequence of human migration from rural areas and ineffective vector and reservoir control [3]–[6]. Canine VL control is based on either treatment or euthanasia of infected animals. However, treatment of canine leishmaniasis with drugs successfully used for human VL shows low efficacy and induces the development of parasitic resistance to these drugs [7]–[10]. The WHO thus strongly recommends that the same drugs should not be used for treatment of dogs and humans in a same area [2]. On the other hand, euthanasia of infected dogs is often unacceptable for ethical and social reasons. Furthermore, the elimination of infected dogs has shown controversial results in Brazil [11], [12]. These issues led to the search of immunotherapy as a treatment alternative for canine VL. The administration of *L. (L.) infantum chagasi* extracts associated with the conventional chemotherapy of naturally infected dogs resulted in a significant reduction in infectivity [13]. Similar results were observed in dogs infected with *L. (L.)* infantum *chagasi* that displayed a significant parasite burden reduction after treatment with autoclaved *L. (L.) majo*r antigens and heat killed *Mycobacterium vaccae* administered in conjunction with Glucantime [14]. The healing efficacy of some vaccine candidates has also been tested. Treatment of infected dogs with purified *L. (L.) infantum chagasi* LiF2 antigen in combination with Glucantime led to the disappearance of clinical signs and a 100% cure rate [15]. Dogs naturally infected with *L. (L.) infantum chagasi* and treated with the recombinant vaccine Leish-110f formulated with the adjuvant MPL-SE associated with Glucantime showed clinical improvement, parasitological cure and increased survival [16]. Recent data supported the effectiveness of this recombinant vaccine for the treatment of mild cases of canine VL [17]. The immunotherapeutic potential of the Leishmune vaccine alone or in association with chemotherapy for canine VL treatment has also been demonstrated [18]–[20].

A recombinant cysteine proteinase from *L. (L.) infantum chagasi*, rLdccys1 was previously shown to be an useful immunological marker for different VL stages in humans and dogs, and to offer an appropriate diagnostic tool for human and canine VL [21]–[23]. Furthermore, immunization with either rLdccys1 or the gene *Ldccys1*, which encodes the cysteine proteinase, induced significant protection against *L. (L.) infantum chagasi* infection mediated by a predominant Th1 response in a murine model of VL [24]. In that study rLdccys1 was administered with *P. acnes*, a Gram-positive bacillus, known to induce a prevalent Th1 immune response in mice [25], [26]. In earlier studies we used *P. acnes* as an adjuvant to immunize BALB/c mice with native Ldccys1; this resulted in a predominant Th1 response and a significant protection against *L. (L.) infantum chagasi* challenge [27]. These results encouraged us to evaluate the immunotherapeutic potential of rLdccys1 plus *P. acnes* for naturally infected dogs from Teresina, Piauí, a state in Brazil with a high incidence of VL [28].

## Methods

### Ethics statement

This study was carried out in strict accordance with the recommendations in the Guide for the Care and Use of Laboratory Animals of the Brazilian National Council of Animal Experimentation (http://www.cobea.org.br). The protocol was approved by the Committee on the Ethics of Animal Experiments of the Institutional Animal Care and Use Committee at the Federal University of São Paulo (Id # CEP 1540/11).

### Animals

A total of thirty dogs of different breeds, ages and sex were provided by the Zoonosis Control Center in Teresina, Piauí, in Brazil, a VL endemic area. The animals were kept in a thin-screened kennel in the Faculdade NOVAFAPI within Teresina, Piauí state and fed commercially balanced animal food (Cherokee, PRT, Brazil). Drinking water was provided *ad libitum*. Diagnostic procedures were performed in the Department of Parasitology and Microbiology at the Federal University of Piauí. All dogs had been pre-treated with anti-tick, anti-scabies and anti-helminthic drugs and had been vaccinated against Parvovirus, Adenovirus type II, Distemper, rabies (Defensor, Pfizer, EUA), Parainfluenza and Corona viruses and Leptospirosis (Vanguard Plus, Pfizer, EUA). Animals diagnosed with distemper, ehrlichiosis, and babesiosis were excluded from the study. Animals with severe renal failure (creatinine and urea values higher than 2.0 mg/dl and 35 mg/dl, respectively) and pancytopenia (total leucocyte number lower than 6,000 and number per mm^3^ of erythrocytes and platelets lower than 5.5×10^3^ and 200, respectively) were not included in the study. All animals selected for immunotreatment tested positive for leishmaniasis in parasitological, serological and biochemical assays, had typical clinical signs of visceral leishmaniasis and were never treated for canine leishmaniasis. Diagnosis was grounded on positive bone marrow aspirate cultures, and ELISA assays using an *L. (L.) chagasi* amastigote extract and rLdccys1 as antigens. Additional laboratory studies included: complete blood cell counts, biochemical assays (creatinine, urea, alkaline phosphatase, aspartate aminotransferase, alanine aminotransferase, albumin, total protein, glucose, and direct and indirect bilirubin). Clinical parameters evaluated were: alopecia, anorexia, apathy, cachexia, hyperkeratosis, size of lymph nodes, ocular secretion, onychogryphosis, skin lesions and weight loss.

### ELISA assays for dog screening

Microtiter plates (high binding, Costar, Corning Incorporated, Corning, New York, USA) were coated with either 100 ng/well of *L. (L.) infantum chagasi* lysates from amastigotes isolated from spleens of *L. (L.) infantum chagasi*-infected hamsters as described elsewhere [29] or with 200 ng/well of rLdccys1 in coating buffer (0.05 M Na_2_CO_3_/NaHCO_3_, pH 9.6). The plates were incubated overnight at 4°C and then blocked with 5% powdered skim milk in PBS for 1 h. Canine sera were diluted 1∶500, added to the plate and incubated for 2 h at room temperature. After three washes with 0.05% Tween 20 in PBS, peroxidase labeled antibodies specific to canine IgG diluted 1∶500 were added to the plate for 1 h at room temperature. The plates were then washed three times with 0.05% Tween 20 in PBS, and the reaction developed with 0.5 mg/ml *o*-phenylenediamine in 0.05 M sodium citrate, pH 4.5, containing 0.03% H_2_O_2_. The reaction was stopped by adding 4 N H_2_SO_4_, and the absorbance was measured at 492 nm in a Multiskan MS Plate Reader (Labsystems Oy, Helsinki, Finland). The cut-off values were calculated by adding two S.D. values to the mean absorbance of 20 dog normal sera.

### Expression and purification of the recombinant cysteine proteinase (rLdccys1)

The PCR product corresponding to the ORF of the *Ldccys1* gene previously obtained [21] was subcloned into the *Bam* HI and *Eco* RI restriction sites of the pHis-parallel 3 expression vector in frame with an amino-terminal six histidine tag [30]. The recombinant plasmids were used to transform *E. coli* BL21 (DE3). Protein expression was carried out by inoculating 500 ml of LB medium containing 100 µg/ml ampicillin with a 25 ml overnight bacterial culture. The suspension was kept in a rotatory shaker at 37°C until reaching log phase (Abs 600 nm = 0.6), and protein expression was then induced with 0.2 mM IPTG for a further 3 h at 37°C. After growth, the recombinant bacteria were pelleted at 4,000× *g* for 10 min, and the recombinant antigen was then purified from the insoluble inclusion bodies by affinity chromatography using a Ni-NTA Superflow agarose matrix (QIAGEN), according to Skeiky et al. [31]. Purified protein was analyzed by SDS-PAGE and Western blotting using a previously described monoclonal antibody directed against a cysteine proteinase of 30 kDa from *L. (L.) amazonensis* (MoAb 2E5D3) that cross reacts with an antigen of 30 kDa from *L. (L.) infantum chagasi* amastigotes [27], [32].

### Heat-killed *Propionibacterium acnes* suspension

*P. acnes* was obtained from Instituto Adolfo Lutz, São Paulo, S.P., Brazil and cultured as previously described [33]. Briefly, the bacteria were grown in anaerobic medium (Hemobac, Probac, São Paulo, S.P., Brazil) for 3 days at 37°C and washed by centrifugation. The resulting pellets were suspended in 0.9% saline and subjected to continuous water vapor for 20 min at 120°C. The protein concentration of the suspension was determined by the Bradford method [34].

### Dog treatment

The treatment protocol was performed with 30 mongrel dogs selected by the parameters described in the ‘Animals’ section. All selected dogs were positive for leishmaniasis in parasitological, serological and biochemical assays and displayed clinical signs of VL. After selection they were randomly divided into three groups of 10 dogs each: one group received three subcutaneous doses of 500 µg rLdccys1 plus 500 µg *P. acnes* as an adjuvant on the back at a one month interval; the second group received three doses of *P. acnes* alone; and the third group received PBS. Half of the dogs from each group were followed by monthly clinical examinations until natural death, while the other half were euthanized three months after the end of treatment.

### Clinical evaluation

The development of the clinical signs of VL was described by a score that quantifies the number of signs and discriminates mild from severe signs. The scoring system for clinical signs was based on the diameters of small (score = 1), and large lymph nodes (score = 2). For mild and severe weight loss were given scores of 3 and 4, respectively. For alopecia, onychogryphosis, cachexia, apathy, anorexia, hyperkeratosis, ocular secretion and skin lesions were attributed a score of 3. The dogs were evaluated at time zero, when they received the first dose, and monthly thereafter until two months after the end of treatment (month 4). Clinical evaluation was performed by a veterinarian which was blinded to the treatment groups evaluated. Animals were looked for side effects of the recombinant antigen such as local pain, local swelling, vomit and diarrhoea. These signs were followed for 10 days after each antigen injection.

### Determination of parasite burden

Parasite burden was evaluated in the spleens of all dogs enrolled in the study immediately after animal death using the limiting dilution assay, as previously described [35], and parasite numbers were determined from the highest dilution at which promastigotes could be grown. Briefly, spleens were aseptically excised, weighed and approximately 1–1.5 g of spleen tissue was collected from the mid area and minced into small pieces with sterile scissors within a sterile Petri dish. The tissue was homogenized in 1 ml of PBS and further diluted in 199 medium (Gibco) containing 4.2 mM NaHCO_3_, 40 mM HEPES, 1.0 mM adenine, 5 µg/ml hemin, 15% fetal bovine serum and 2% male human urine to obtain a final concentration of 1 mg/ml. Serial dilutions ranging from 1 to 1×10^−6^ were prepared in the same medium under sterile conditions in 96 wells micro plates (Costar plates; Corning, Inc., Corning, NY). After incubation for 10 days at 26°C, plates were examined using an optical microscope at 3-day intervals. The reciprocal of the highest dilution that was promastigote positive was considered to be the concentration of parasites in the spleen tissue processed and the total parasite load was calculated by multiplying this value by the total spleen weight.

### Evaluation of immune responses

Delayed-type hypersensitivity assays (DTH) were performed by intradermal injection of rLdccys1 (10 µg) into the inner surface of the right thigh. As a negative control, each animal received an injection of PBS into the inner surface of the left thigh. The induration diameter was measured by use of a caliper after 24, 48 and 72 h, and each time the values of the saline control were subtracted from the reaction due to the rLdccys1 antigen. Skin reactions with diameter equal or larger than 5 mm were considered positive. The DTH assays were carried out at time zero and one month after animals received the third dose of rLdccys1.

Specific anti-rLdccys1 antibodies IgG, IgG1 and IgG2 isotypes were evaluated by ELISA at time zero and one month after administration of each dose of rLdccys1 in microtiter plates coated with 200 ng/well of rLdccys1 according to the protocol described in “ELISA assays for dog screening” section except that canine sera were diluted 1∶200. Peroxidase labeled antibodies specific to canine IgG were diluted 1∶500 and to IgG1 or IgG2 isotypes (Bethyl Laboratories, Inc., Montgomery, TX, USA) were diluted 1∶2000 and added to the plate for 1 h at room temperature. The plates were then washed and the reaction developed as described above.

Lymphokine concentrations were measured in dog sera at time zero and one month after administration of each dose of rLdccys1 using a double-sandwich ELISA assay (Quantikine Canine IFNγ and IL-10) (R&D Systems). Microtiter plates (high-binding Costar plates; Corning, Inc., Corning, NY) were coated overnight at 4°C with specific mAb, which was directed to each lymphokine tested and used at 100 ng/well. After washing with 0.05% Tween 20/PBS (PBS/T) and blocking with PBS/T containing 5% skim milk for 2 h at 37°C, 100 µl of dog sera diluted 1∶2 were added to wells. Standard curves were generated using recombinant canine IFNγ and IL-10. After incubation overnight at 37°C, plates were washed with PBS/T, and a second antibody specific to each lymphokine was added (biotinylated antibody diluted 1∶250). After 60 min at 37°C, the plates were washed three times in 0.05% Tween 20 in PBS, and the reaction was developed with 0.5 mg/ml of *o*-phenylenediamine in 0.05 M sodium citrate, pH 4.5, containing 0.03% H_2_O_2_. The reaction was stopped by adding 4N H_2_SO_4_, and the absorbance was measured at 492 nm in a Multiskan Plate Reader (Labsystems Oy, Helsinki, Finland). Serum concentrations higher than the minimal values obtained from the respective standards were considered to be positive.

### Statistical analysis

One-way ANOVA and Student's t-test were used to determine the significant differences between groups by use of GraphPad Prisma (version 5.0) and *P* values smaller than 0.05 (*P*<0.05) were considered significant. The Pearson correlation coefficient was also calculated by use of GraphPad Prisma (version 5.0).

## Results

### 1) Reactivity of rLdccys1 in delayed–type hypersensitivity (DTH) and humoral responses

**Figure 1:**
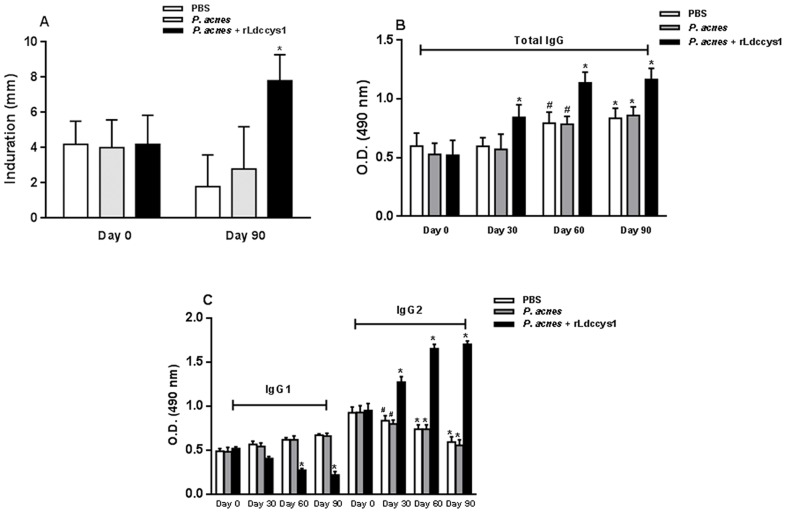
Delayed–type hypersensitivity and humoral responses induced by *L. (L.) chagasi* rLdccys1 in dogs treated with the recombinant antigen compared with controls. (A) DTH evaluated at time zero and one month after the third dose of rLdccys1 (day 90) (n = 10 animals per group). *P*<0.001; (B and C) – Total IgG (B) and IgG1 and IgG2 (C) were evaluated by ELISA assay in the sera of treated dogs at time zero and one month after each dose of rLdccys1. Plates were sensitized with 200 ng/well of rLdccys1 and the sera from all of the dogs (n = 10 animals per group) were collected 30 days after each dose and diluted 1∶200. *P<0.001; ^#^ *P*<0.05.

Figure 1A shows that at time zero, selected dogs from each of the groups exhibited low DTH values, whereas one month after the end of treatment the DTH values were significantly higher in dogs that received rLdccys1 compared to controls. The production of total IgG at time zero and one month after administration of each dose of rLdccys1 is shown in Figure 1B, while dosages of IgG1 and IgG2 are illustrated in Figure 1C. Starting from the first dose, there was a significant increase of IgG production in the sera of dogs treated with the recombinant antigen. Starting from the second dose, there was also an increase of IgG production in sera from control dogs, although this increase was lower than that observed in animals treated with rLdccys1. The characterization of IgG subclasses showed that there was a significant increase of IgG2 in animals treated with rLdccys1, whereas IgG1 production was significantly reduced after the second and third doses. In contrast, there was constant production of IgG1 in controls during all treatment time and a significant reduction of IgG2 starting from the first dose.

### 2) Determination of lymphokine production

**Figure 2:**
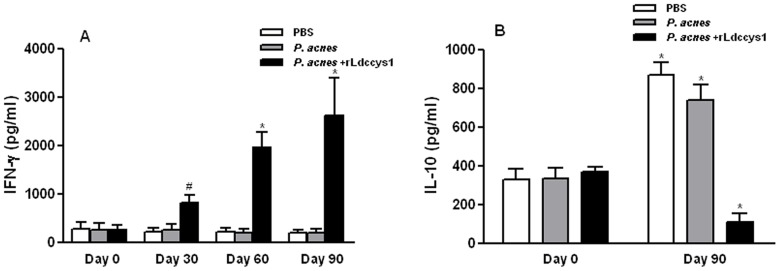
Levels of IFN-γ (A) and IL-10 (B) in dog sera. (A) IFN-γ was quantified at time zero and one month after each dose of rLdccys1. (B) IL-10 was quantified at time zero and one month after the third dose of rLdccys1 (n = 10 animals per group) ^*^ *P*<0.001; ^#^ *P*<0.05.

IFN-γ and IL-10 concentrations were measured by ELISA in dog sera. Figure 2 shows that one month after the first dose there was a low but significant IFN-γ secretion in animals treated with the recombinant antigen. Furthermore, an increased concentration of this lymphokine was observed after the second and third doses of rLdccys1, while a low IL-10 concentration was detected in these animals. In contrast, the control dogs exhibited a low IFN-γ concentration in all periods analyzed, as well as a significant IL-10 increase after they received the third dose of either *P. acnes* alone or saline. These data indicate that the treatment with the recombinant antigen led to the activation of Th1 responses.

### 3) Clinical evaluation

**Figure 3:**
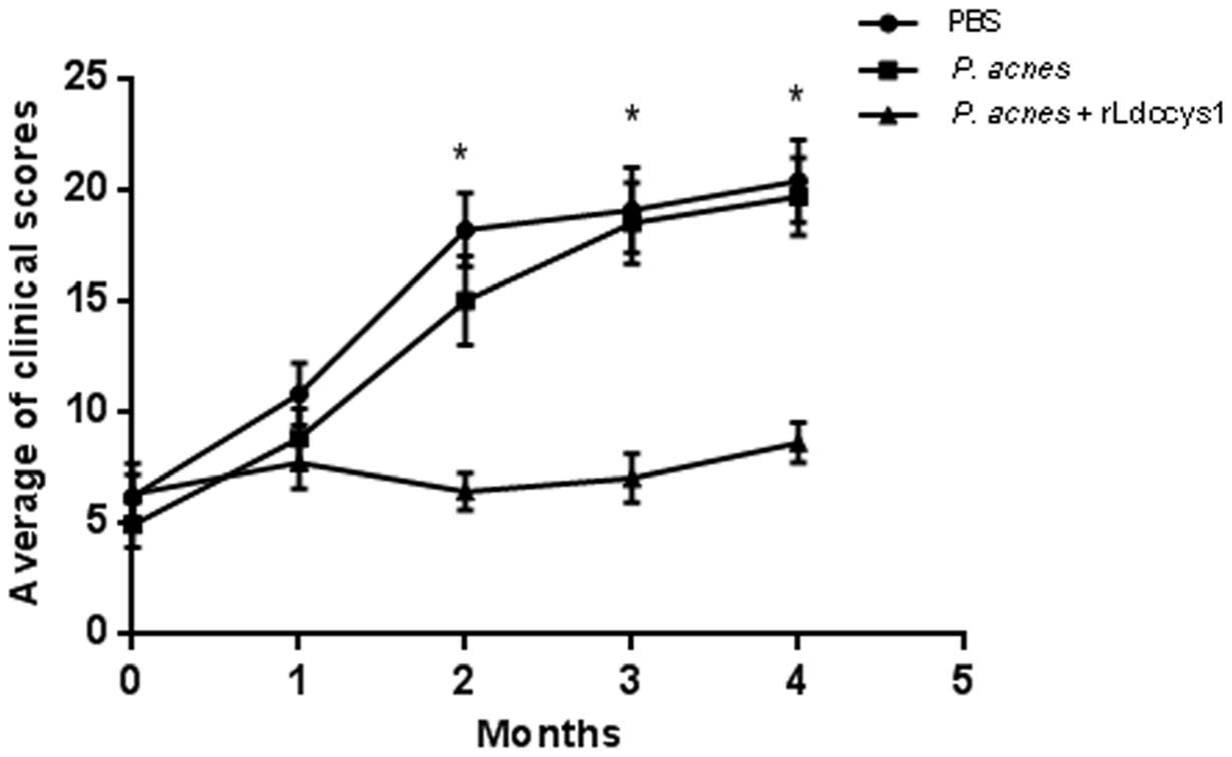
Clinical evaluation. Outcome of clinical scores in controls and dogs treated with rLdccys1. Each point represents the average of clinical scores from treated dogs that were evaluated on time zero until two months following the end of treatment (n = 10 animals per group). ^*^ *P*<0.001.

The evolution of clinical signs of VL is shown in Figure 3 and Table S1. At time zero, animals from the three groups exhibited a similar clinical score average (saline, 6.2; *P. acnes*, 4.9; rLdccys1 plus *P. acnes*, 6.3). Control animals exhibited a significant increase of clinical signs that indicate disease progression two months after the end of treatment. In contrast, there was no increase of clinical signs in dogs treated with rLdccys1, indicating that they were able to control the disease development. It is also important to emphasize that clinical signs implicated in the severity of canine VL, such as weight loss, cachexia and anorexia, were observed only in control dogs (Table S1).

The safety of the recombinant antigen was also evaluated. The number of dogs showing pain (25%) increased significantly from the first to the third dose (*P*<0.001). The pain after each treatment dose lasted for 48 h. Local swelling was the most common adverse effect observed (67%) reaching an average diameter of 5 cm and no significant differences among doses 1, 2 and 3 were noted. Furthermore, in all dogs the local swelling reaction was transient and decreased 24 h after each dose, disappearing five days after injection. Dogs did not vomit or present diarrohea.

**Figure 4:**
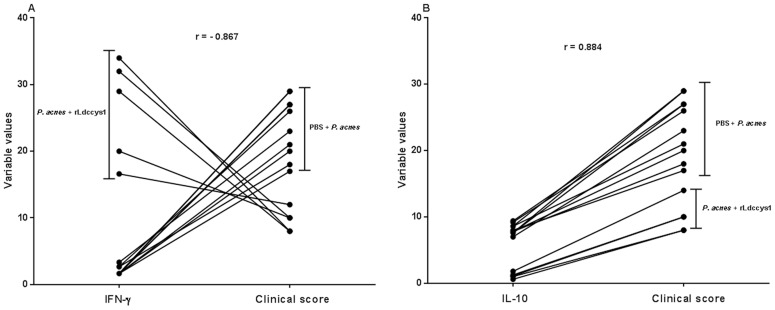
Correlation between lymphokine production and the average of clinical scores in controls and treated dogs one month after treatment with rLdccys1. Data are depicted in a bivariate correlation graph between individual clinical signs and levels of IFN-γ (A) and IL-10 (B) in controls (PBS or *P. acnes*) and dogs treated with rLdccys1. *P*<0.001.

The correlation between lymphokine production and clinical score averages 30 days after the end of treatment with rLdccys1 is shown in Figure 4. The correlation between the production of IFN-γ and the clinical score averages is negative because there was a significant increase of IFN-γ followed by low clinical scores in dogs treated with rLdccys1, whereas in the controls the low secretion of IFN-γ was correlated with an increase in clinical signs (Figure 4A). In contrast, there is a direct correlation between IL-10 production and the clinical score average. A significant increase in IL-10 and clinical score averages was observed in controls, while there was a decrease in IL-10 secretion followed by low clinical scores in dogs treated with rLdccys1 (Figure 4B).

### 4) Evaluation of the dog survival and parasite load

**Figure 5:**
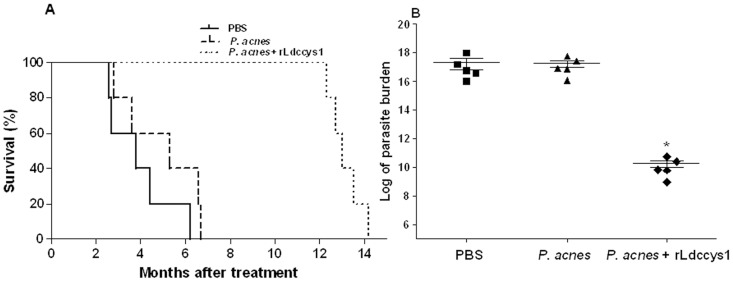
Survival (A) and parasite load (B) of controls and dogs treated with rLdccys1. The parasite load was evaluated by limiting dilution analysis in the spleen of the dogs immediately after animal death. *P<0.05. Bars represent the standard error of the mean and horizontal lines represent the average of parasite load. The standard deviations are 3.782e+017, 2.108e+017, and 2.256e+010, for PBS, *P. acnes*, and *P. acnes* + rLdccys1 groups, respectively.

The survival of controls and dogs treated with rLdccys1 was followed until the natural death of half of the *L. (L.) chagasi*-infected animals. Most of the dogs that received saline died between 2.6 and 4.4 months after the end of treatment. Among these animals, one was observed to have the lowest clinical score average and survived until 6 months after treatment. Among the dogs treated with *P. acnes*, three died between 2.8 and 5.3 months, while two of them survived 6.6 and 6.7 months after treatment. In contrast, dogs treated with rLdccys1 died after 12.3 to 14.2 months, with a mean survival time two times higher than the controls (Figure 5A). Figure 5B shows the parasite load evaluated by limiting dilution analysis in dog spleens after death. Animals treated with rLdccys1 exhibited a seven-log reduction in parasite burden compared to controls that received either saline or *P. acnes* alone.

The other half of the dogs that were euthanized three months after the end of treatment showed a lower parasite burden compared to animals followed until natural death. However, the parasite burden reduction in rLdccys1-treated dogs was not significantly different between euthanized and non-euthanized animals (data not shown).

## Discussion

The main problems that face the treatment of leishmaniasis are toxicity, high cost and parasitic resistance to leishmanicidal drugs currently available on the market. This scenario is even more pronounced in the case of canine VL due to the low efficacy of the drugs currently in use for treatment of infected dogs [36]. All of these issues have pointed to the immunological stimulation as an attractive option for the treatment of leishmaniasis [37]. With this rationale, the potential of a recombinant cysteine proteinase of *L. (L.) infantum chagasi*, rLdccys1, for the treatment of canine VL was investigated in this study. The usefulness of this recombinant antigen was first demonstrated in screening naturally infected dogs selected for this study. Similar values were observed when either rLdccys1 or *L. (L.) infantum chagasi* extracts were used as antigens in ELISA assays for evaluation of humoral responses (data not shown). These findings corroborate previous results that demonstrated the high sensitivity and specificity of rLdccys1 for the diagnosis of canine and human VL [21], [23]. In this study, a significant increase of DTH responses was observed after treatment with rLdccys1, supporting our previous results on the use of rLccys1 to distinguish between asymptomatic and symptomatic canine VL [23].

Data on antibody production showed an increase of total IgG in all animal groups during the treatment; however this increase was higher in the rLdccys-treated dogs. Furthermore, rLdccys1-treated dogs had increased IgG2 production and decreased IgG1, while control animals exhibited lower antibody levels with predominance of IgG1. Although the IgG1 and IgG2 subclasses have been used as more reliable indicators of the CVL status than total IgG [38], recently, the functional characterization of canine IgG subclasses raised doubts about the correlation of IgG subclasses with Th1 or Th2 responses [39]. However, our findings are compatible with those that showed high levels of IgG1 anti-*Leishmania* antibodies associated with the development of clinical signs in *L. (L.) infantum chagasi*-infected dogs, while IgG2 antibodies appear to be associated with asymptomatic infection [40]. Furthermore, protective responses in dogs vaccinated with the recombinant A2 protein appear to be associated with increased levels of total IgG and IgG2 but not with those of IgG1 anti-A2 antibodies [41]. Our data on DTH and humoral responses in rLdccys1-treated dogs are also compatible with those reported in dogs infected with *L (L.) infantum chagasi* subjected to immunotherapy with the Leishmune vaccine [18], [20].

The treatment with rLdccys1 resulted in a significant increase of IFN-γ, reduction in IL-10, and significantly less progressive clinical disease than control groups. In contrast, the control dogs presented the opposite profile of these lymphokines and a significant increase of clinical signs until four months after treatment. Indeed, a significant negative correlation was found between the number of clinical signs and IFN-γ production in dogs treated with rLdccys1, whereas a positive correlation was observed between the production of IL-10 and an increase of clinical signs in control dogs. These results strengthen the implication of IFN-γ and IL-10 in control and progression, respectively of canine VL. The induction of Th1 cells producing IFN-γ, IL-2 and TNF-α has been associated with protection against canine VL [42], [43]. The activation of macrophages by IFN-γ to kill intracellular amastigotes via the L-arginine nitric oxide pathway is the main effector mechanism involved in the protective immune responses of dogs infected with *L. (L.) chagasi infantum* [44], [45]. In contrast, IL-10 has been correlated with disease progression and an increase in IL-10 levels; this was observed in the spleens of dogs naturally infected with *L. (L.) infantum chagasi* [46]. IL-10 mRNA transcripts were detected in Con A-stimulated PBMC derived from dogs with VL clinical signs [43], [47]. It is worth noting that among the rLdccys1-treated dogs, the first animal that died exhibited a higher clinical score mean at screening, as well as a lower level of serum IFN-γ one month after the end of treatment (data not shown). These findings indicate that the effectiveness of the rLdccys1 treatment is dependent on the disease progression at the time of inclusion in the study. It is possible that sick animals with lower levels of IL-10 respond better to antigen stimulation. Similar results were observed in dogs developing severe VL that did not respond to Leish-111f vaccine treatment [17]. These considerations point to the treatment of asymptomatic dogs with rLdccys1 and a potential more pronounced Th1 response in these animals.

It is also important to emphasize the choice of the adjuvant used in the present study. *P. acnes* treatment elicits a type-1 (Th1) immune response involving IL-12 and IL-18 that induces IFN-γ release, enhancement of the IgG2a switch and Th2 expansion inhibition [25], [26]. Administration of killed *P. acnes* as an adjuvant increased the resistance to infection by *Trypanosoma cruzi* [48]. In leishmaniasis, the treatment with *P. acnes* led to the control of *L. (L.) major* infection in BALB/c mice [49]. Murine vaccination with the A2 antigen from *L. (L.) donovani* plus *P. acnes* resulted in a mixed Th1 and Th2 response with a predominance of Th1 responses after the homologous challenge, as well as a significant parasite burden decrease in immunized animals [50]. Our previous data on immunization of BALB/c mice with either the native or recombinant Ldccys1 plus *P. acnes* followed by challenge with *L. (L.) infantum chagasi* also showed a predominant Th1 response and a significant parasite burden decrease in immunized animals [24], [27]. Immunization of dogs with rLdccys1 plus *P. acnes* also resulted in a significant protection against *L. (L.) infantum chagasi* infection (unpublished data).

The participation of CD8^+^ lymphocytes in protective immune responses triggered in dogs treated with rLdccys1 was not addressed in this study but cannot be overlooked. Analysis of the predicted amino acid sequence of the *L. (L.) infantum chagasi Ldccys1* gene cloned previously showed one potential MHC class I epitope for CD8^+^ lymphocytes in addition to two MHC class II epitopes [21]. The involvement of CD8^+^ lymphocytes in canine VL has been demonstrated [51] and increased levels of these cells appear to be the major phenotypic feature of asymptomatic disease [52]. Enhanced expression of CD8^+^ lymphocytes was also observed in *L. (L.) infantum chagasi*-infected dogs after treatment with the Leishmune vaccine and resulted in a significant reduction of VL clinical signs and parasite burden levels [18], [20].

Immunotherapy with rLdccys1 increased the survival time of *L. (L.) infantum chagasi*-infected dogs. Comparable results were reported following the use of Glucantime and the recombinant Leish-110f vaccine for treatment of dogs naturally infected with *L. (L.) infantum chagasi* [16]. Contrary to our findings, animals subjected to that immunotherapeutic protocol had a reduced number of deaths. Nevertheless, treated animals were followed until 180 days after treatment, while in our study animals were monitored until their natural death, and all of the rLdccys1-treated dogs were alive up until one year after treatment. Use of the Leish-110f vaccine in infected dogs treated for longer period of time also resulted in lower death rates compared to our results [17]. Again, this study was different from our treatment protocol because the authors emphasized the advantage of a weekly vaccine schedule over antigen administration with 3 or 4 weeks intervals [17]. Higher survival rates were also observed in dogs treated with the Leishmune vaccine [18]–[20]. It is important to highlight that the differences among previous studies on immunochemotherapy and the present study, including treatment schedules, adjuvant, geographical factors, and disease status of the dogs, hinder the comparison with our data. It is still important to mention that only symptomatic dogs with a high parasite burden were selected for our study and presumably treatment with rLdccys1 of animals with lower parasite burden may lead to improved results.

Despite the high parasitism levels found within the infected dogs enrolled in the present study, there was a significant lesser parasite burden in dogs treated with rLdccys1 compared to controls. Nevertheless, dog cure and a pronounced decrease of vector infectivity are desirable goals in regions of high VL endemicity. In this context, we believe that the use of booster doses of rLdccys1 associated to allopurinol, the drug recommended by WHO to treat CVL [2], are promising to improve the effectiveness of treating CVL with this recombinant antigen.

In conclusion, our findings showed the potential of rLdccys1 as an additional tool for immunotherapy of canine VL and support further studies to evaluate its healing efficacy with a larger number of dogs, as well as in different regions of VL incidence.

## Supporting Information

Table S1 **Clinical scores of control and rLdccys1-treated dogs with time.** The clinical scores of all dogs enrolled in the study, as well as the average of clinical scores of the three groups are shown.
(DOC)
