# Supplementary material for: Use of a Recombinant Cysteine Proteinase from Leishmania (Leishmania) infantum chagasi for the Immunotherapy of Canine Visceral Leishmaniasis
Source: PLoS Negl Trop Dis. 2014 Mar 13;8(3):e2729. doi: 10.1371/journal.pntd.0002729 (PMC3953064; doi:10.1371/journal.pntd.0002729)
Supplement: Table S1 — Clinical scores of control and rLdccys1-treated dogs with time. The clinical scores of all dogs enrolled in the study, as well as the average of clinical scores of the three groups are shown. (DOC) [file pntd.0002729.s001.doc]

**Table S1. Clinical scores of control and rLdccys1-treated dogs with time**

| **Group** | **Dog** | | | **MONTHS** | | | | | | | | | |
| --- | --- | --- | --- | --- | --- | --- | --- | --- | --- | --- | --- | --- | --- |
|  |  |  |  | 0 | | 1 | | 2 | | 3 | | 4 | |
| **PBS** | **1** | | 7 | | 14 | | 20 | | 23 | | 21 | |  |
|  |  | | O,H,L1 | | O,Sl,H,Os ,L2 | | O,Ap,Lp,H,Os,L2,W3 | | A,O,C,Ap,H,Os,L2,W3 | | O,C,Ap,H,Os,L2,W4 | |  |
|  | **2** | | 10 | | 14 | | 26 | | 29 | | 29 | |  |
|  |  | | O,H,Os,L1 | | O,H,Os,L2,W3 | | A,O,An,Ap,Lp,H,Os,L2,W3 | | A,O,C,An,Ap,Sl,H,Os,L2,W3 | | A,O,C,An,Ap,Sl,H,Os,L2,W3 | |  |
|  | **3** | | 11 | | 15 | | 23 | | 27 | | 27 | |  |
|  |  | | A,O,H,L2 | | A, O,H,L2,W4 | | A,O,An,Ap,H,Os,L2,W3 | | A,O,C,An,Ap,H,Os,L2,W4 | | A,O,C,An,Ap,H,Os,L2,W4 | |  |
|  | **4** | | 10 | | 14 | | 13 | | 20 | | 26 | |  |
|  |  | | O,Sl,H,L1 | | O,Sl,H,L2,W3 | | O,Lp,H,L1,W3 | | A,O,C,Sl,H,L2,W3 | | A,O,C,,Ap,Sl,H,Os,L2,W3 | |  |
|  | **5** | | 4 | | 5 | | 23 | | 15 | | 18 | |  |
|  |  | | O,L1 | | O,L2 | | A,O,Ap,Lp,H,Os,L2,W3 | | O,Sl,H,L2,W4 | | A,O,Sl,H,L2,W4 | |  |
|  | **6** | | 4 | | 5 | | 18 | | 18 | | 18 | |  |
|  |  | | O, L1 | | O,L2 | | A,O,Lp,H,L2,W4 | | A,O,Sl,H,L2,W4 | | A,O,Sl,H,L2,W4 | |  |
|  | **7** | | 5 | | 14 | | 15 | | 15 | | 15 | |  |
|  |  | | O,,L2 | | A,O,H,L2,W3 | | A,O,H,L2,W4 | | A,O,H,L2,W4 | | A,O,H,L2,W4 | |  |
|  | **8** | | 4 | | 5 | | 11 | | 11 | | 14 | |  |
|  |  | | A,L1 | | O,L2 | | O,Lp,H,L2 | | O,H,L2,W3 | | A,O,H,L2,W3 | |  |
|  | **9** | | 5 | | 14 | | 21 | | 21 | | 24 | |  |
|  |  | | O,L2 | | O,Sl,H,L2,W3 | | A,O,Lp,H,Os,L2,W4 | | A,O,Sl,H,Os,L2,W4 | | A,O,Ap,Sl,H,Os,L2,W4 | |  |
|  | **10** | | 2 | | 8 | | 12 | | 12 | | 12 | |  |
|  |  | | L2 | | H,L2,W3 | | O,H,L2,W4 | | O,H,L2,W4 | | O,H,L2,W4 | |  |
| **Average**  **of scores** | | | **6,2** | | **10,8** | | **18,2** | | **19,1** | | **20,4** | |  |
| ***P.acnes*** | **1** | | 10 | | 13 | | 26 | | 27 | | 27 | |  |
|  |  | | O,H,Os,L1 | | O,Sl,H,Os,L1 | | O,C,An,Ap,Sl,H,Os,L2,W3 | | O,C,An,Ap,Sl,H,Os,L2,W4 | | O,C,An,Ap,Sl,H,Os,L2,W4 | |  |
|  | **2** | | 10 | | 14 | | 26 | | 29 | | 29 | |  |
|  |  | | O,Sl,Os,L1 | | O,Sl,Os,L2, W3 | | O,C,An,Ap,Sl,H,Os,L2,W3 | | A,O,C,An,Ap,Sl,H,Os,L2,W3 | | A,O,C,An,Ap,Sl,H,Os,L2,W3 | |  |
|  | **3** | | 7 | | 14 | | 17 | | 20 | | 20 | |  |
|  |  | | A,O,L1 | | A,O,Sl,H,L2 | | A,O,Sl,H,Os,L2 | | A,O,Sl,H,Os,L2,W3 | | A,O,Sl,H,Os,L2,W3 | |  |
|  | **4** | | 4 | | 7 | | 11 | | 17 | | 23 | |  |
|  |  | | O,L1 | | O,L1,W3 | | O,H,Os,L2 | | A,O,H,Os,L2,W3 | | A,O,Ap,Sl,H,Os,L2,W3 | |  |
|  | **5** | | 2 | | 5 | | 14 | | 20 | | 17 | |  |
|  |  | | L2 | | O,L2 | | A,O,Ap,L2,W3 | | A,O,Ap,Sl,H,L2,W3 | | A,O,Sl,H,L2,W3 | |  |
|  | **6** | | 5 | | 11 | | 11 | | 15 | | 18 | |  |
|  |  | | O,L2 | | O,H,L2,W3 | | O,H,L2,W3 | | A,O,H,L2,W4 | | A,O,Sl,H,L2,W4 | |  |
|  | **7** | | 2 | | 5 | | 8 | | 11 | | 11 | |  |
|  |  | | L2 | | Sl,L2 | | A,Sl,L2 | | A,Sl,L2,W3 | | A, Sl,L2,W3 | |  |
|  | **8** | | 4 | | 10 | | 11 | | 14 | | 17 | |  |
|  |  | | O,L1 | | A,O,H,L1 | | A,O,H,L2 | | A,O,H,L2,W3 | | A,O,H,Os,L2,W3 | |  |
|  | **9** | | 1 | | 2 | | 15 | | 18 | | 21 | |  |
|  |  | | L1 | | L2 | | A,O,H,L2,W4 | | A,O,Sl,H,L2,W4 | | A,O,Sl,H,Os,L2,W4 | |  |
|  | **10** | | 4 | | 7 | | 11 | | 14 | | 14 | |  |
|  |  | | O,L1 | | A,O,L1 | | A,O,H,L2 | | A,O,H,L2,W3 | | A,O,H,L2,W3 | |  |
| **Average**  **of scores** | | | **4,9** | | **11,8** | | **15,0** | | **18,5** | | **19,7** | |  |
| **rLdccy1+**  ***P. acnes*** | | **1** | 7 | | 7 | | 5 | | 5 | | 8 | |  |
|  |  |  | O,Sl,L1 | | O,Sl,L1 | | O,L2 | | O,L2 | | A,O,L2 | |  |
|  |  | **2** | 4 | | 7 | | 5 | | 4 | | 8 | |  |
|  |  |  | O,L1 | | O,Sl,L1 | | O,L2 | | O,L1 | | O,H,L2 | |  |
|  |  | **3** | 14 | | 16 | | 11 | | 14 | | 14 | |  |
|  |  |  | O,Ap,H,Os,L2 | | O,Sl,H,Os,L1,W3 | | O,Sl,Os,L2 | | O,Sl,Os,L2,W3 | | O,Sl,Os,L2,W3 | |  |
|  |  | **4** | 5 | | 7 | | 8 | | 10 | | 10 | |  |
|  |  |  | O,L2 | | O,L1,W3 | | A,O,L2 | | O,Ap,L1,W3 | | O,Ap,L1,W3 | |  |
|  |  | **5** | 14 | | 10 | | 5 | | 7 | | 10 | |  |
|  |  |  | O,Sl,H,Os,L2 | | O,Sl,L1,W3 | | O,L2 | | O,L1,W3 | | O,Sl,L1,W3 | |  |
|  |  | **6** | 5 | | 11 | | 11 | | 11 | | 11 | |  |
|  |  |  | O,L2 | | O,H,Os,L2 | | A,O,H,L2 | | A,O,H,L2 | | A,O,H,L2 | |  |
|  |  | **7** | 1 | | 4 | | 4 | | 4 | | 4 | |  |
|  |  |  | L1 | | O,L1 | | O,L1 | | O,L1 | | O,L1 | |  |
|  |  | **8** | 5 | | 5 | | 5 | | 5 | | 5 | |  |
|  |  |  | O,L2 | | O,L2 | | O,L2 | | O,L2 | | O,L2 | |  |
|  |  | **9** | 4 | | 5 | | 5 | | 5 | | 8 | |  |
|  |  |  | O,L1 | | O,L2 | | O,L2 | | O,L2 | | O,H,L2 | |  |
|  |  | **10** | 4 | | 5 | | 5 | | 5 | | 8 | |  |
|  | |  | O,L1 | | O,L2 | | O,L2 | | O,L2 | | O,H,L2 | |  |
| **Average**  **of scores** | | | **6,3** | | **7,7** | | **6,4** | | **7,0** | | **8,6** | |  |

Dogs were evaluated at screening (month 0), one month after first (month 1), second (month 2) and third (month 3) doses and 2 months after the end of treatment (month 4) for: alopecia (A), anorexia (An), apathy (Ap), cachexia (C), hyperkeratosis (H), lymph node size (L), onychogryphosis (O), ocular secretion (Os), skin lesion (Sl), weight loss (W).

Alopecia, anorexia, apathy, cachexia, hyperkeratosis, onychogriphosis, ocular secretion and skin lesion were considered of value=3. Weight loss was classified as 3 when ranging from 0-2 kg, and 4 for severe weight loss when ranging from 2-5 kg. To lymph node enlargement were attributed values of 1for medium (≤1.9 cm) and 2 for large (≥2 cm).
